# *GSTO1*, *GSTO2* and *ACE2* Polymorphisms Modify Susceptibility to Developing COVID-19

**DOI:** 10.3390/jpm12030458

**Published:** 2022-03-14

**Authors:** Tatjana Djukic, Goran Stevanovic, Vesna Coric, Zoran Bukumiric, Marija Pljesa-Ercegovac, Marija Matic, Djurdja Jerotic, Nevena Todorovic, Milika Asanin, Marko Ercegovac, Jovan Ranin, Ivana Milosevic, Ana Savic-Radojevic, Tatjana Simic

**Affiliations:** 1 Faculty of Medicine, University of Belgrade, 11000 Belgrade, Serbia; tatjana.djukic@med.bg.ac.rs (T.D.); goran_drste@yahoo.com (G.S.); vesna.coric@med.bg.ac.rs (V.C.); zoran.bukumiric@med.bg.ac.rs (Z.B.); marija.pljesa-ercegovac@med.bg.ac.rs (M.P.-E.); marija.matic@med.bg.ac.rs (M.M.); djurdja.jovanovic@med.bg.ac.rs (D.J.); masanin@gmail.com (M.A.); ercegovacmarko@gmail.com (M.E.); njranin@gmail.com (J.R.); ivana.milosevic00@gmail.com (I.M.); 2 Institute of Medical and Clinical Biochemistry, 11000 Belgrade, Serbia; 3 Clinic of Infectious and Tropical Diseases, Clinical Centre of Serbia, 11000 Belgrade, Serbia; nevena.todorovic.1992@gmail.com; 4 Institute of Medical Statistics and Informatics, 11000 Belgrade, Serbia; 5 Clinic of Neurology, Clinical Centre of Serbia, 11000 Belgrade, Serbia; 6 Clinic of Cardiology, Clinical Centre of Serbia, 11000 Belgrade, Serbia; 7 Department of Medical Sciences, Serbian Academy of Sciences and Arts, 11000 Belgrade, Serbia

**Keywords:** oxidative distress, COVID-19, glutathione transferase omega, ACE2, polymorphisms

## Abstract

Based on the close relationship between dysregulation of redox homeostasis and immune response in SARS-CoV-2 infection, we proposed a possible modifying role of *ACE2* and glutathione transferase omega (*GSTO*) polymorphisms in the individual propensity towards the development of clinical manifestations in COVID-19. The distribution of polymorphisms in *ACE2* (rs4646116), *GSTO1* (rs4925) and *GSTO2* (rs156697) were assessed in 255 COVID-19 patients and 236 matched healthy individuals, emphasizing their individual and haplotype effects on disease development and severity. Polymorphisms were determined by the appropriate qPCR method. The data obtained showed that individuals carrying variant GSTO1*AA and variant GSTO2*GG genotypes exhibit higher odds of COVID-19 development, contrary to ones carrying referent alleles (*p* = 0.044, *p* = 0.002, respectively). These findings are confirmed by haplotype analysis. Carriers of H2 haplotype, comprising GSTO1*A and GSTO2*G variant alleles were at 2-fold increased risk of COVID-19 development (*p* = 0.002). Although *ACE2* (rs4646116) polymorphism did not exhibit a statistically significant effect on COVID-19 risk (*p* = 0.100), the risk of COVID-19 development gradually increased with the presence of each additional risk-associated genotype. Further studies are needed to clarify the specific roles of glutathione transferases omega in innate immune response and vitamin C homeostasis once the SARS-CoV-2 infection is initiated in the host cell.

## 1. Introduction

Initially affecting the lungs, pandemic coronavirus disease 2019 (COVID-19) caused by severe acute respiratory syndrome coronavirus 2 (SARS-CoV-2) represents a complex multisystem disease with significant variations in severity, duration and outcomes. Multifaceted interrelationships between inflammation, hypoxia, disrupted redox homeostasis and activity of redox-sensitive signaling pathways have emerged as important aspects of COVID-19 development and progression. It has been well documented that oxidative distress can also affect entry and fusion of SARS-CoV-2 into the host cell, as well as its replication [1].

The omega glutathione transferase (GSTO) class, characterized by a unique set of enzymatic and metabolic functions, exhibits an important role in redox regulation, glutathionylation cycle and innate immune response [2]. Both GSTO1-1 and GSTO2-2 isoforms have a cysteine residue in their active site (Cys-32), allowing them to catalyze different types of reactions, including thioltransferase and dehydroascorbate reductase activities [3,4]. Studies on patients with chronic obstructive pulmonary disease (COPD) showed that extracellular GSTO1-1 may modulate glutathione (GSH) homeostasis and antioxidant defense in airway secretions [5]. What is more, it was shown that GSTO1-1 can modulate the proinflammatory lipopolysaccharide (LPS)/Toll-like receptor (TLR-4)-induced activation of the nuclear factor kappa B (NF-κB) pathway in macrophages [6]. Furthermore, a small molecule inhibitor of GSTO1-1 was recently shown to attenuate the inflammatory response to LPS [7]. All these findings brought the researchers to the conclusion that GSTO1-1 can exert a pro-inflammatory action on innate immunity. As the enzyme with the highest dehydroascorbate-reductase (DHAR) activity in humans, GSTO2-2 is responsible for maintaining high cellular vitamin C levels and is involved in the regulation of redox homeostasis and proper function of the immune system as well. 

So far, several studies have reported that a high-dose intravenous vitamin C had positive effects on the treatment of moderate to severe COVID-19 patients due to its potential inhibitory effect on SARS-CoV-2 multiplication [8]. In the recent genome-wide association analyses, the significance of GSTO2 in pulmonary function was also recognized, but its precise role and function in the lungs is still unclear [9]. Several polymorphisms exist for both human *GSTO1* and *GSTO2* genes. Two commonly studied single nucleotide polymorphisms are: GSTO1*C419A (rs4925) and GSTO2*A424G (rs156697) [10]. In the *GSTO1* rs4925 polymorphism, the variation results from a cytosine (C) to adenine (A) transition at base 419 and causes an alanine (Ala) to aspartic acid (Asp) substitution at the position 140 (Ala140Asp). As for the *GSTO2* rs156697 polymorphism, the variation results from a adenine (A) to guanine (G) transition at base 424 and causes an asparagine (Asn) to aspartic acid (Asp) substitution at position 142 (Asn142Asp) [4]. It has been shown that the *GSTO1* rs4925 polymorphism causes changes primarily in deglutathionylase activity [11,12,13]. Namely, Menon and Board have shown that the variant GSTO1*A allele has lower deglutathionylase activity and higher activity in the forward glutathionylation reaction, in contrast to the GSTO1*C wild-type allele [11,12]. Regarding the *GSTO2* rs156697 polymorphism, a strong association between variant GSTO2*G alleles and lower *GSTO2* gene expression has been shown [14,15]. Despite the fact that many recent papers suggest the involvement of oxidative distress in SARS-CoV-2 infection, data on the glutathione transferase omega (GSTO) class are lacking.

On the other hand, SARS-CoV-2 enters the host cells via endocytosis following the interaction of viral S-protein and angiotensin converting enzyme 2 (ACE2) after priming by tissue-specific transmembrane serine proteases, including TMPRSS2 [16]. The initial findings imply that genetic variants in the ACE2 protein can modify the predisposition to infection of a person exposed to SARS-coronavirus-2 [17]. Specifically, the C-terminal receptor binding domain (RBD) of the spike protein binds to human ACE2 through interactions with its N-terminal peptidase M2 domain. In ACE2, 18 amino acid residues located in four regions around the N-terminal alpha-helical lobes were critical for interaction with the RBD domain of SARS-CoV spike protein [18]. Three single-nucleotide variations, including K26R (rs4646116) had a higher binding affinity than the referent *ACE2* and greater susceptibility to SARS-CoV-2 infection [19]. The rs4646116 polymorphism (rs4646116 SNP report. Available online: https://www.ncbi.nlm.nih.gov/snp/rs4646116?horizontal_tab=true#variant_details (accessed on 8 February 2022)), representing one of the most common missense variants, results from a thymine (T) to cytosine (C) transition encoding the replacement of Lys (K) to Arg (R) at position 26, which is positioned immediately beside the ACE2-Spike protein interface [20].

In view of significant variations in response to SARS-CoV-2 infection and the supposed role of oxidative distress, inter-individual differences in severity of clinical manifestations in COVID-19 patients might be affected by their *GSTO* and *ACE2* genetic profile. What is more, glutathione transferases omega 1 and omega 2 with their glutathionylase and dehydroascorbate-reductase activities may influence the patients’ inclination to more severe forms of COVID-19. Therefore, the aim of our study was to assess the distribution of genetic polymorphisms in genes encoding *ACE2* (rs4646116), *GSTO1* (rs4925) and *GSTO2* (rs156697) in COVID-19 patients.

## 2. Materials and Methods

The study was conducted in accordance with principles of the International Conference on Harmonization (ICH) Good Clinical Practice as well as the “Declaration of Helsinki”, and was previously approved by the Ethics Committee of the Clinical Centre of Serbia (number 566/01 from 13 July 2020 and number 608/01 from 7 August 2020).

This case-control study recruited 255 patients (mean age 52.00 ± 12.6 years, 54.5% males) from Institute of Infectious and Tropical Diseases, University Clinical Centre of Serbia, between July 2020 and February 2021, diagnosed with COVID-19 by means of a positive RT-PCR test, as previously described [21]. A detailed insight into the genomic characterization of SARS-CoV-2 whole genome sequences from the second wave of the epidemic in Serbia, taking place from June 2020, was reported in the recent study by Miljanovic et al. [21]. From October 2020 to February 2021, the presence of 20A, 20B, 20C, 20D, 20E (EU1), 20G, 20I/Alpha clades of SARS-CoV-2 were detected with different frequencies throughout these months. During this period, the highest frequency was proven to be 20D and 20A clades. The first cases of British strain (20I/Alpha) were detected in December 2020, with the increased frequency of this strain in the following months (SARS-CoV-2 whole-genome sequences are available in GISAID database, manuscript under preparation, personal communication with Professor A.Knezevic, on 25 February 2022). Patients were stratified according to the COVID-19 National Guidelines, version 9, into those with mild COVID-19 (Stage I) and severe COVID-19 (Stage II, III and IV). The case group did not comprise patients treated within ICUs or those with lethal outcomes. The control subjects were initially selected by the absence of detectable SARS-CoV-2 antibodies (IgM and IgG) and further on matched according to age and gender, comprising 236 individuals (mean age 50.00 ± 14.14 years, 53.8% males). Written informed consent was acquired from all recruited participants. Clinical, demographic and epidemiological data were collected using the RedCap^®^ based questionnaire (RedCap^®^. Available online: https://redcap.med.bg.ac.rs/, AntioxIdentification (accessed on 8 February 2022)).

Blood samples were collected in EDTA-coated test tubes for DNA isolation (PureLink™ Genomic DNA Mini Kit, ThermoFisher Scientific, Waltham, MA, USA). *GSTO1* (rs4925, ID number: C__11309430_30), *GSTO2* (rs156697, ID number: C___3223136_1_), *ACE2* (rs4646116, ID number:C__32336201_30) polymorphisms were determined by TaqMan Drug Metabolism Genotyping assays (Life Technologies, Applied Biosystems, Waltham, MA, USA) on a Mastercycler ep realplex platform (Eppendorf, Hamburg, Germany). Other laboratory parameters were procured from routine laboratory practice.

Numerical data were presented by measures of central tendency (arithmetic mean and median) and measures of variability (standard deviation and interquartile range), whereas attributive data were presented in absolute and relative numbers. Statistical analysis comprised testing statistical hypotheses with appropriate tests using IBM SPSS Statistics 22 (IBM Corporation, Armonk, NY, USA) software. Based on normality tests, the differences in continuous data with non-normal distribution were assessed using Kruskal–Wallis test. Differences between categorical variables, as well as Hardy-Weinberg equilibrium for respective genotypes, were tested for using a χ2-test. The risk for COVID-19 development and progression was calculated with adjusted odds ratios (OR) and 95% confidence intervals (CI) using logistic regression analysis.

## 3. Results

Baseline characteristics of the study group comprising 491 participants are given in Table 1. As presented, COVID-19 patients did not differ significantly from the control group in terms of age and gender (*p* > 0.05). The presence of hypertension, diabetes or obesity was associated with higher risk of COVID-19 development. Concisely, individuals with hypertension had 2-fold higher risk of COVID-19 development (OR = 2.15, 95%CI: 1.41–3.26, *p* < 0.001) than normotensive subjects. The presence of diabetes was associated with a 2-fold higher risk of COVID-19 development as well (OR = 2.15, 95%CI: 1.02–4.04, *p* = 0.043). The highest risk of COVID-19 was observed in subjects with BMI < 30 (OR = 2.87, 95%CI: 1.81–4.57, *p* < 0.001). Conversely, smoking was associated with a decreased risk of COVID-19 development (OR = 0.22, 95%CI: 0.14–0.35, *p* < 0.001).

Differences in terms of hematologic, biochemical, coagulation and inflammatory biomarkers between patients with mild and severe forms of COVID-19 are presented in Table 2. Patients exhibiting a severe form of COVID-19 had lower counts of lymphocytes (*p* < 0.001), monocytes (*p* = 0.019) and platelets (*p* = 0.033) than patients with mild COVID-19. The severe form of COVID-19 was accompanied with lower levels of Fe (*p* = 0.002) and TIBC (*p* = 0.039) as well, whereas levels of other biochemical parameters were higher than in patients with mild COVID-19: AST (*p* < 0.001), LDH (*p* < 0.001), urea (*p* = 0.005) and creatinine (*p* = 0.009). Additionally, patients with severe COVID-19 had higher levels of D-dimer (*p* = 0.046). All examined inflammatory biomarkers (CRP, fibrinogen, serum ferritin and IL-6) were higher in patients with severe COVID-19 (*p* < 0.001) as well.

The distribution of *GSTO1* (rs4925), *GSTO2* (rs156697) and *ACE2* (rs4646116) genotypes among COVID-19 patients and controls adjusted for age, gender, hypertension, diabetes mellitus, smoking and obesity is presented in Table 3. In the analysis of given polymorphisms, both *GSTO1* (rs4925) and *GSTO2* (rs156697) reached statistically significant association with the risk of COVID-19 development. Namely, the odds of COVID-19 development were significantly increased among individuals with variant GSTO1*AA genotype compared to the GSTO1*CC homozygotes (OR = 2.45 95%CI: 1.03–5.84, *p* = 0.044). Additionally, the risk of COVID-19 increased with the presence of each additional variant GSTO2*G allele: GSTO2*AG carriers were in almost 2-fold higher odds of COVID-19 development (OR = 1.91, 95%CI = 1.10–3.30, *p* = 0.020), while GSTO2*GG homozygotes exhibited 3.7-fold higher odds of COVID-19 development (OR = 3.69, 95%CI = 1.62–8.40, *p* = 0.002). *ACE2* (rs4646116) polymorphism did not exhibit a statistically significant effect on COVID-19 risk, although the carriers of ACE2*TC + CC genotype were at slightly increased risk in comparison to individuals with ACE2*TT genotype (OR = 1.79, 95%CI = 0.89–3.57, *p* = 0.100).

Since both *GSTO1* and *GSTO2* genes are located on the same chromosome, the influence of their polymorphisms on the risk of COVID-19 development was further analyzed by a haplotype analysis (Table 4). Normalized linkage disequilibrium coefficient of LD (D’) between *GSTO* polymorphisms was 0.6113, *p* = 0.0043 confirming a high LD between these pairs of SNPs. Haplotype H1 (consisted of GSTO1*C (rs4925) and GSTO2*A (rs156697) referent alleles) represented a reference group for analysis, since it was the most prevalent haplotype among controls (66%) and patients (56%). The second most frequent haplotype was H2, comprised of variant alleles of both examined *GSTO* polymorphisms: GSTO1*A (rs4925) and GSTO2*G (rs156697). According to our results, carriers of H2 haplotype were at the highest risk of COVID-19 development (OR = 1.97, 95%CI: 1.28–3.03, *p* = 0.002).

Based on the identified COVID-19 risk-associated genotypes, their cumulative effect on the disease development was further analyzed (Table 5). The combination of genotypes exhibiting the lowest odds of COVID-19 development (GSTO1*CC/GSTO2*AA/ACE2*TT) was denoted as a Reference category. According to our results, the risk of COVID-19 development gradually increased with the presence of each additional risk-associated genotype: OR = 1.45, 95%CI = *p* = 0.248 in individuals with one risk-associated genotype; OR = 2.50, *p* = 0.004 in individuals with two risk-associated genotypes; and OR = 3.03, *p* = 0.064 in those with three risk-associated genotypes.

The cumulative effect of *GSTO* and *ACE2* genotypes was also analyzed in terms of the disease severity. Namely, grouping patients with mild symptoms vs. severe symptoms enabled us to evaluate the prognostic potential of the suggested risk-associated genotype combination, but no statistical significance was found (Table 6).

## 4. Discussion

Based on the close relationship between dysregulation of redox homeostasis and immune response in SARS-CoV-2 infection, we proposed a possible modifying role of *ACE2* and glutathione transferase omega (*GSTO*) polymorphisms in individual propensity towards the development of clinical manifestations in COVID-19. Indeed, the data obtained showed that individuals carrying variant GSTO1*AA (rs4925) and variant GSTO2*GG (rs156697) genotypes exhibit higher odds of COVID-19 development, contrary to ones carrying referent alleles. These findings are further confirmed by the haplotype analysis. Carriers of H2 haplotype, comprising GSTO1*A and GSTO2*G variant alleles, revealed 2-fold increased odds of COVID-19 development. Although, the *ACE2* (rs4646116) polymorphism did not exhibit a statistically significant effect on COVID-19 risk, the risk of COVID-19 development gradually increased with the presence of each additional risk-associated genotype.

To the best of our knowledge, this is the first study addressing the association of *GSTO1* and *GSTO2* polymorphisms with COVID-19 development. It seems that glutathione transferase omega 1-1 (GSTO1-1) isoenzyme, beyond its catalytic activity, also exhibits a role in innate immune response. This important pleiotropic role in both redox regulation and innate immunity appears to be interconnected primarily with its function in the glutathionylation cycle. Namely, as a posttranslational modification involved in redox regulation, glutathionylation of proteins impacts different cellular processes, including inflammation [22]. In this context, an important role of GSTO1-1 in promoting activation of NLRP3 inflammasome has been recently demonstrated [23]. NLRP3 inflammasome represents one of the most important innate immune components and is involved in the conversion of the cytokine precursors, pro-IL-1β and pro-IL-18, into mature IL-1β and IL-18. This process results in the subsequent inflammatory cascade, supporting the release of additional NLRP3 cytokines, such as IL-6 [24]. Recent findings on the deglutathionylation of NIMA related kinase 7 (NEK7) by GSTO1 as a necessary event in NLRP3 inflammasome activation further supports the significance of this enzyme in the first phase of antiviral host defense [25]. However, it is important to note that abnormal activation of NLRP3 inflammasome is frequently associated with tissue injury during infection. So far, several studies have reported hyperactivation of the NLRP3 inflammasome in COVID-19, as well as its relation to pathologies associated with severe COVID-19 such as acute respiratory distress syndrome (ARDS) and acute lung injury (ALI) [24,26].

In this study, we investigated how polymorphisms in GSTO1-1, as an enzyme involved in activation of the NLRP3 inflammasome, modify risk of COVID-19. It has been shown that the GSTO1*C allele has higher deglutathionylation activity in contrast to the GSTO1*A variant allele [11,12]. It reasonable to speculate that the presence of *GSTO1* allelic variants with altered deglutathionylation activity and lower activation of NEK7/NLRP3 inflammasome could provide a probable mechanism to explain the associations between this genetic polymorphism and innate immune response. Additionally, Piaggi et al. found that soluble GSTO1-1 in the airways of cystic fibrosis patients correlated with inflammatory parameters such as neutrophilic elastase and IL-8. Furthermore, the variant GSTO1*AA was associated with lower levels of the anti-inflammatory mediators prostaglandin E2 (PGE2) and 15-(S)-hydroxyeicosatetraenoic acid (15(S)-HETE) [27]. Thus, our results on the association between the variant GSTO1*AA (rs4925) genotype and increased susceptibility towards COVID-19 development seem biologically plausible.

In addition to GSTO1, the SNP polymorphism of the *GSTO2* gene could also affect primarily its antioxidant dehydroascorbate reductase (DHAR) activity (Whitbread et al., 2005; Piacentini et al., 2013), which is responsible for regeneration of ascorbic acid (vitamin C). Having in mind beneficial effects of vitamin C on COVID-19 patients [8], low DHAR activity in patients with both variant *GSTO2* alleles may result in deficient vitamin C regeneration and accumulation of the oxidized form, dehydroascorbate, contributing to disruption of redox homeostasis. In this setting, inefficient regeneration of vitamin C, as a potential result of the *GSTO2* polymorphism, may also contribute to activation of hypoxia-inducible factor-1α (HIF-1α). One of the main enzymes involved in marking HIF-1α for ubiquitination and proteasomal degradation depends on vitamin C as a cofactor [28]. In this context, it may be hypothesized that ascorbic acid-dependent inhibition of HIF signaling provides an additional approach to controlling inflammation [29]. As a possible consequence of *GSTO2* polymorphisms, diminished regeneration of ascorbic acid may also influence HIFα hydroxylation and promote its accumulation. Indeed, recent data imply an important role of HIF-1α in promoting SARS-CoV-2 infection and inducing pro-inflammatory responses to COVID-19 [29]. In this context, it can be speculated that vitamin C-dependent inhibition of the HIF-1α pathway may provide an additional approach to controlling inflammation [29]. Assuming the specific roles of GSTO enzymes in these processes, our results support the hypothesis that GSTO polymorphisms are associated with the risk of COVID-19, with special emphasis on the *GSTO2*-variant genotype.

Although it has been reported that polymorphisms in the *ACE2* gene may play an important role in the pathogenesis of COVID-19 using 3D modeling, potential modifying effects on COVID-19 development have not been studied in clinical settings until now [30]. In regard to the investigated *ACE2* polymorphism, we observed higher odds for COVID-19 development in carriers of the variant *ACE2* genotype, who exhibited a higher binding affinity than those of the referent *ACE2* genotype and obviously greater susceptibility to SARS-CoV-2 infection; however, the difference was not statistically significant. A significant contribution to redox homeostasis perturbations caused by SARS-CoV-2 infection is from viral replication as well as the way the virus takes advantage of the human redox system for its own needs. Each of these steps has the potential to affect function and activity of redox-sensitive targets. Under oxidative stress, the extracellular environment becomes oxidation-prone, resulting in more disulfide formation on protein surfaces [31]. Therefore, under severe oxidative stress, the cell surface receptor ACE2 and receptor binding domain of the intruding viral spike protein are likely to be present in their oxidized form, having predominantly disulfide linkages. Hati and Bhattacharyya have shown that under oxidative stress, the lack of a reducing environment results in significantly favorable binding of the viral protein on the cell surface receptor ACE2 [32].

## 5. Conclusions

Overall, our results on the association between *GSTO* genetic variants and COVID-19 development further imply the significance of these enzymes in the regulation of redox homeostasis and immune response, especially inflammasome activation. By improving our knowledge of COVID-19 genetics and the association between individual phenotype and genotype data, as well as the underlying mechanisms, we will be in a position to improve management systems and tailor therapy in order to ensure better patient care.

## Figures and Tables

**Table 1:** Baseline characteristic of COVID-19 patients and respective controls.

Baseline Characteristic	COVID-19 Patients (*n* = 255)	Controls (*n* = 236)	OR (95%CI)	*p*
Age (years) ^a^	52.00 ± 12.67	50.00 ± 14.14	/	0.100
Gender, *n* (%)				
Male	139 (54.5)	127 (53.8)	1.00 ^b^	
Female	116 (45.5)	109 (46.2)	0.97 (0.68–1.39)	0.877
Hypertension, *n* (%) ^c^				
No	98 (54.1)	152 (71.7)	1.00 ^b^	
Yes	83 (45.9)	60 (28.3)	2.15 (1.41–3.26)	<0.001
Obesity, *n* (%) ^c^				
BMI < 30	159 (64.4)	161 (83.9)	1.00 ^b^	
BMI > 30	88 (35.6)	31 (16.1)	2.87 (1.81–4.57)	<0.001
BMI (kg/m^2^) ^a^	28.82 ± 5.25	26.09 ± 4.29	/	<0.001
Smoking, *n* (%) ^c^				
Never	133 (54.5)	88 (37.8)	1.00 ^b^	
Former	73 (29.9)	31 (13.3)	1.56 (0.95–2.56)	0.082
Ever	38 (15.6)	114 (48.9)	0.22 (0.14–0.35)	<0.001
Diabetes, *n* (%) ^c^				
No	228 (89.4)	223 (94.5)	1.00 ^b^	
Yes	27 (10.6)	13 (5.5)	2.03 (1.02–4.04)	0.043

^a^ Mean ± SD; ^b^ Reference group; ^c^ Percentage.

**Table 2:** Hematologic, biochemical, coagulation and inflammatory biomarkers in patients with mild and severe forms of COVID-19.

Biomarkers ^a^	Mild COVID-19 (*n* = 82)	Severe COVID-19 (*n* = 169)	Reference Values	*p*
**Hematologic**				
WBC count (10^9^/L)	5.62 ± 2.30	6.21 ± 2.54	3.4–9.7	0.121
Lymphocyte count (10^9^/L)	1.61 ± 0.62	1.35 ± 1.34	1.2–3.4	<0.001
Monocyte count (10^9^/L)	0.62 ± 0.45	0.48 ± 0.28	0.10–0.80	0.019
Platelet count (10^9^/L)	219.98 ± 59.25	205.15 ± 77.46	158–424	0.033
RBC count (10^12^/L)	4.81 ± 0.44	4.70 ± 0.54	3.86–5.08	0.213
Hemoglobin (g/L)	141.56 ± 12.55	135.55 ± 23.75	119–157	0.063
**Biochemical**				
ALT (U/L)	45.54 ± 31.44	53.67 ± 34.04	0–41	0.073
AST (U/L)	28.00 ± 15.40	41.30 ± 24.36	0–37	<0.001
LDH (U/L)	212.78 ± 113.83	301.89 ± 193.77	220–460	<0.001
Urea (mmol/L)	4.83 ± 1.78	6.17 ± 4.86	2.5–7.5	0.005
Creatinine (µmol/L)	77.95 ± 16.65	92.80 ± 47.15	45–84	0.009
Fe (µmol/L)	10.31 ± 6.43	6.07 ± 4.05	7.0–28.0	0.002
TIBC (µmol/L)	61.56 ± 63.84	43.25 ± 10.57	44.8–75.1	0.039
**Coagulation**				
D-dimer (mg/L)	0.57 ± 0.43	1.36 ± 6.44	<0.5	0.046
**Inflammatory biomarkers**				
CRP (mg/L)	15.36 ± 34.33	65.22 ± 64.05	0.0–5.0	<0.001
Fibrinogen (g/L)	3.10 ± 0.86	4.32 ± 1.46	2.0–4.0	<0.001
Serum ferritin (µg/L)	293.94 ± 394.02	707.39 ± 740.24	13.0–150.0	<0.001
IL-6 (pg/mL)	9.90 ± 17.78	40.93 ± 44.28	<7	<0.001

^a^ Mean ± SD.

**Table 3:** The distribution of genotypes among COVID-19 patients and controls.

Genotype	COVID-19 Patients *n*, %	Controls *n*, %	Adjusted OR (95%CI) ^a^	*p*
*GSTO1* (*rs4925*)				
CC (AlaAla)	132 (52.4)	126 (54.3)	1.00 ^b^	
CA (AlaAsp)	78 (31.0)	88 (37.9)	1.42 (0.83–2.42)	0.205
AA (AlaAsp)	42 (16.7)	18 (7.8)	2.45 (1.03–5.84)	0.044
*GSTO2* (*rs156697*)				
AA (AsnAsn)	115 (45.1)	137 (60.1)	1.00 ^b^	
AG (AsnAsp)	89 (34.9)	74 (32.5)	1.91 (1.10–3.30)	0.020
GG (AspAsp)	51 (20.0)	17 (7.5)	3.69 (1.62–8.40)	0.002
*ACE2* (*rs4646116*)				
TT (LysLys)	195 (77.7)	179 (89.5)	1.00 ^b^	
TC + CC (LysArg + ArgArg)	56 (22.3)	21 (10.5)	1.79 (0.89–3.57)	0.100

^a^ OR, odds ratio adjusted for gender, age, hypertension, diabetes mellitus, smoking and obesity; CI, confidence interval; ^b^ Reference group.

**Table 4:** Haplotypes of *GSTO1* (rs4925) and *GSTO2* (rs156697) in relation to the risk of COVID-19.

Haplotype	*GSTO1* rs4925	*GSTO2* rs156697	Controls %	COVID 19 Patients %	Adjusted OR (95%CI) ^a^	*p*
H1	*C	*A	66.13	56.37	1.00 ^b^	
H2	*A	*G	16.75	26.18	1.97 (1.28–3.03)	0.002
H3	*C	*G	7.15	11.28	1.95 (1.00–3.81)	0.050
H4	*A	*A	9.97	6.18	0.97 (0.48–1.95)	0.920

Global haplotype association *p*-value = 0.0043 for adjusted analysis; ^a^ OR, odds ratio adjusted for gender, age, hypertension, diabetes mellitus, smoking and obesity; CI, confidence interval; ^b^ Reference group. * Allele.

**Table 5:** Cumulative effect of COVID-19 risk-associated genotypes.

Number of Risk-Associated Genotypes	COVID-19 Patients *n* (%)	Controls *n* (%)	OR (95%CI) ^a^	*p*
**0**	72 (29.0)	82 (41.4)	1 ^b^	
**1**	67 (27.0)	61 (30.8)	1.45 (0.77–2.73)	0.248
**2**	86 (34.7)	50 (25.3)	2.50 (1.34–4.64)	0.004
**3**	23 (9.3)	5 (2.5)	3.03 (0.94–9.78)	0.064

0: reference genotype combination carrying lowest odds for COVID-19: (GSTO1*CC/GSTO2*AA/ACE2*TT); 1, 2, 3: the number of risk-associated genotypes (either one, two or three COVID-19 risk-associated genotypes), comprising GSTO1*CA + AA or GSTPO2*AG + GG or ACE2*TC + CC; ^a^ OR, odds ratio adjusted for age, gender, hypertension, diabetes mellitus, smoking and obesity; ^b^ Reference group; CI, confidence interval.

**Table 6:** The association of risk-associated genotypes with risk for severe COVID-19.

Number of Risk-Associated Genotypes	Mild COVID-19 Patients *n* (%)	Severe COVID-19 Patients *n* (%)	OR (95%CI) ^a^	*p*
**0**	19 (24.1)	53 (29.5)	1 ^b^	
**1**	21 (26.6)	44 (26.6)	0.58 (0.18–1.92)	0.371
**2**	31 (39.2)	53 (34.4)	1.07(0.37–3.06)	0.906
**3**	8 (10.1)	15 (9.4)	0.93 (0.19–4.50)	0.926

Mild COVID-19: Stage I; Severe COVID-19: Stages II + III + IV. 0: reference genotype combination carrying lowest odds for severe COVID-19 (GSTO1*CC/GSTO2*AA/ACE2*TT); 1, 2, 3: The number of risk-associated genotypes (either one, two or three risk-associated genotypes), comprising GSTO1*CA + AA or GSTPO2*AG + GG or ACE2*TC + CC; ^a^ OR, odds ratio adjusted for age, gender, hypertension, diabetes mellitus, smoking and obesity; ^b^ Reference group; CI, confidence interval.

## Data Availability

The data supporting reported results are available at *RedCap* platform (Research Electronic Data Capture, Vanderbilt University) of Faculty of Medicine University in Belgrade and will be made available by the corresponding authors upon request without undue reservation.
